# Otology

**Published:** 1905-04-29

**Authors:** 


### OTOLOGY.

**The Treatment of Purulent Otitis Media.**—Wm. Milligan[1] quotes the results recently published by Schmiegelow in the treatment of 110 cases of purulent otitis. In 61 of these a cure was effected without complications supervening. Two cases, complicated with diabetes, ended fatally; in five cases a dry perforation persisted; and in six the suppurative process became chronic. Schmiegelow is strongly in favour of paracentesis as soon as the diagnosis is made. In 71 cases in which that operation was performed, the length of time the discharge persisted was greater the later the date of the operation. Thus when paracentesis was performed between the first and third days the average duration of the disease was 11 days, whereas when performed after the 10th day the duration was two months. After paracentesis the cases were treated by the daily insertion of a wick of iodoform gauze soaked in a 2-per-cent. carbolic solution. No irrigation was employed.

**Errors in the Diagnosis and Treatment of Ear Disease** have been discussed by J. Dundas Grant[2] in a clinical lecture. There is a form of desquamative external otitis in which layer after layer of epidermic scales is thrown off from the membrana tympani and meatus. These scales undergo fatty degeneration and form a plug blocking up the meatus. The plug is called an external cholesteatoma. It is often mistaken for cerumen, but it is lighter in colour and more irregular on the surface. Syringing makes the plug swell. Drops composed of 10 grains of salicylic acid, 3 drams of rectified spirit, and 5 drams of glycerine will soften the masses and enable one to remove them with forceps. Epithelioma of the external meatus may be easily mistaken for ordinary granulations, *e.g.* on the posterior wall of the meatus. It is a mistake to attempt to remove foreign bodies in the ear with forceps. Many such bodies will come away with syringing, but others will not. If instrumental removal is attempted an anæsthetic should be given. A fine rectangular sharp-pointed instrument may sometimes be insinuated beyond the body and used to roll it out. Redness and swelling in the mastoid region may be due to a furuncle in the meatus, and not to mastoiditis. For incising such furunculi the author recommends a minute knife with the point directed towards the operator. The cut is made outwards with this, and there is no fear of damaging deeper structures. Politzerisation is sometimes useful for distinguishing between otitis externa and otitis media. In acute otitis media carbolised glycerine drops, 1 in 20, used warm, are far better than warm oil and other remedies not of an antiseptic nature. When an acute suppuration of the middle ear goes on for six weeks it is fairly certain that there is suppuration in the mastoid antrum and cells, and these should be opened. In attic suppuration the neck of the malleus may become soldered to the wall of the attic and fixed. The dulness of hearing thus produced is not remedied by Politzerisation, and if the membrana tympani is relaxed the inflation makes matters worse. A relaxed membrane may be made more tense by treatment with pure collodion applied on a probe armed with cotton wool. If there is catarrh of one ear, a "sympathetic nerve deafness" in the other ear may result. The catarrhal ear only should be inflated in such cases. In cases of cholesteatoma of the tympanum watery drops should not be used, as they cause the cholesteatomatous material to swell up. On the other hand, alcoholic drops may give considerable relief. It is a mistake to use pilocarpine in the treatment of nerve deafness associated with anæmia.

**A Modification of the Radical Mastoid Operation.**—Charles J. Heath[3] describes a method of performing the radical mastoid operation which differs considerably from that adopted by other surgeons. The alleged advantages of the method are: (1) Diminution of shock, as the operation can be performed in half-an-hour; (2) no disfiguring scar; (3) no shaving of the scalp; (4) the after-treatment is easy for both patient and surgeon; (5) the surgeon can discover the direction in which the antrum lies and its depth before removing any bone; and (6) a considerable amount of hearing is restored and preserved. Only a few short hairs near the top of the ear are cut. A square rubber-sheet cap is used to cover the scalp, the ear being passed through a hole near one side. The cap is fixed with forceps over the forehead. The incision, which is 1¼ to 1½ inches long, begins just above the upper attachment of the pinna and runs back in the angle where the cartilage joins the scalp as far as the bottom of the bony meatus. The cartilaginous meatus is defined in its upper and back parts, and also the margin of the bony meatus and the post-meatal spine. The upper and back part of the cartilaginous meatus is separated, together with the skin continuous with it, as far as the drum membrane. Special probes are now used to find the antrum, which is then opened with chisel and mallet. Long burrs are next used to make the cavity smooth. The bridge over the iter is removed with a chisel, and then the malleus and incus are removed. The mucous membrane of the attic is removed, but, if the Eustachian tube is in good order, the mucous membrane of the lower part of the tympanum, including that over the oval window, is left. The meatus is incised along the top from just within the concha to the tympanum, the incision being joined, at its external end, by another incision made at right angles to it and carried backwards and downwards to the middle of the floor of the meatus. A flap is thus formed and turned backwards and downwards to cover the floor and part of the posterior wall of the newly-enlarged meatus. The edge of the cartilage is secured to the edge of the pericranium with cat-gut. A drainage-tube as large as will go in is inserted, and the ear replaced and sutured in its natural position. A wet dressing is applied. In the after-treatment no syringing is employed; spirit drops are recommended after the first two or three days, and exuberant granulations, if they arise, are rubbed with a 50 per cent. nitrate of silver solution. The author lays great emphasis on the fact that his operation preserves hearing power.

[1] Pract., Nov. 1904, p. 687. [2] Clin. Jour., Oct. 19, 1904, p. 9 [3] Lancet, Dec. 24, 1904, p. 1767.

(*To be continued.*)

